# The efficacy of topical treatments for acanthosis nigricans: a systematic review of randomized controlled trials

**DOI:** 10.3389/fmed.2025.1641322

**Published:** 2025-10-10

**Authors:** Awadh Alamri, Rose A. Alraddadi, Dhaii Alzahrani, Amal H. Abualola, Hadeel A. Maaddawi, Renad F. Alharthy, Alanoud Y. Alkhashan, Maria Y. Ashqan, Esraa A. Shaheen, Bashaer Almahdi, Hatoon M. Althobaiti

**Affiliations:** ^1^King Abdullah International Medical Research Center, Jeddah, Saudi Arabia; ^2^Division of Dermatology, Department of Medicine, Ministry of the National Guard-Health Affairs, Jeddah, Saudi Arabia; ^3^College of Medicine, King Saud Bin Abdulaziz University for Health Sciences, Jeddah, Saudi Arabia; ^4^Emergency Medicine, East Jeddah General Hospital, Jeddah, Saudi Arabia; ^5^Department of Dermatology, College of Medicine, King Saud University, Riyadh, Saudi Arabia; ^6^Family Medicine, Ministry of Health, Jeddah, Saudi Arabia; ^7^Employee Health Clinic, King Abdulaziz Medical City, Jeddah, Saudi Arabia; ^8^Intensive Care Unit, King Salman Bin Abdulaziz Medical City, Almadinah Almunawara, Saudi Arabia

**Keywords:** acanthosis nigricans, urea, tretinoin, hyperkeratosis, hyperpigmentation, erythema, keratolytic agents, glycolic acid

## Abstract

**Background:**

Acanthosis nigricans (AN) is a skin disorder marked by darkening and thickening of the skin, often linked to metabolic abnormalities. This systematic review of randomized controlled trials (RCTs) assesses the comparative effectiveness and tolerability of different topical treatment options of AN, aiming to determine the most suitable therapeutic strategies.

**Methods:**

A comprehensive literature search was conducted across PubMed, Scopus, ClinicalTrials.gov, Web of Science, and the Cochrane Library, yielding 6,407 studies. After screening and a full-text review, seven randomized controlled trials (*n* = 268) assessed topical urea (10–20%), tretinoin (0.025–0.05%), salicylic acid (10%), and chemical peels such as glycolic acid (35–70%) and trichloroacetic acid (15%) over 8 weeks to 2 months, primarily on the neck and axilla. Outcomes included melanin and erythema indices (M/E), ANASI/ANSC scores, Investigator’s and Participant’s Global Evaluation (IGE/PGE), and adverse events.

**Results:**

Urea demonstrated significant efficacy in reducing erythema, particularly at higher concentrations (20%), with mild adverse events such as stinging or irritation. Tretinoin was the most effective for reducing dark pigmentation, especially on the neck, and patients were more satisfied with it than with glycolic acid. Salicylic acid (10%) gave results similar to urea, with only mild side effects like dryness or peeling. Trichloroacetic acid (15%) peel was more effective than glycolic acid (35%) peel, both in skin improvement and patient satisfaction after 8 weeks. Overall, side effects with all treatments were mild and went away on their own.

**Conclusion:**

Both urea and tretinoin are effective treatments for AN, choice of therapy should be individualized tretinoin for predominant hyperpigmentation, urea for erythema or lower irritation tolerance, salicylic acid as a tolerable alternative, and TCA peel when stronger procedural options are suitable glycolic peel showed more modest effects.

**Systematic review registration:**

Prospero registration number: (CRD42023444441); https://www.crd.york.ac.uk/PROSPERO/view/CRD42023444441.

## Graphical abstract

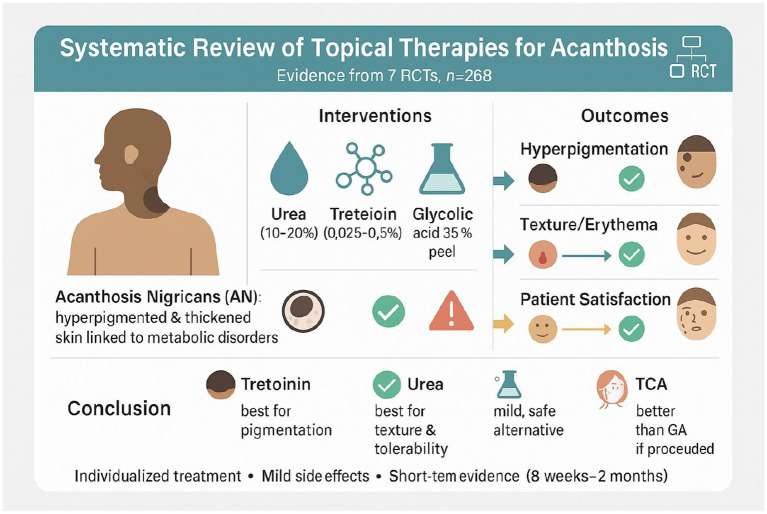

## Introduction

Acanthosis nigricans (AN) is a dermatological condition characterized by darkened, velvet-like skin patches, predominantly found in body folds such as the neck, underarms, and groin areas, as well as occasionally on the outer surfaces of limbs (1, 2). Although generally benign, this skin manifestation may signal underlying systemic conditions, including insulin resistance, obesity, hormonal disturbances, malignancies, and adverse reactions to medications (3, 4). The significance of AN lies in its potential role as a cutaneous marker for metabolic disorders, particularly in patients with obesity and those diagnosed with type 2 diabetes mellitus (T2DM) (5, 6). Global studies have revealed that the prevalence of AN in overweight children and adolescents ranges from 49.2 to 58.2% (7–9). This prevalence is notably higher among patients with severe obesity, those from high-risk ethnic groups, or those already diagnosed with T2DM, underscoring the condition’s association with metabolic abnormalities (10, 11).

Treating AN involves addressing its root cause. For AN linked to obesity, lifestyle changes like weight loss and physical activity enhance insulin sensitivity and decrease hyperinsulinemia, improving skin conditions (12, 13). Pharmaceutical interventions are also crucial, with topical treatments like retinoids, calcipotriol, and TCA peels frequently used (14). In severe cases, systemic therapies, including oral retinoids such as isotretinoin and acitretin, are effective but have potential side effects (15). Metformin, which increases insulin sensitivity, benefits metabolic factors and reduces dermatological signs of AN (16–19). Despite available treatments, varied origins and clinical manifestations of AN make establishing standardized protocols difficult (20). Systemic therapies require careful evaluation of overall health and potential adverse reactions. This highlights the need for a thorough, personalized approach to managing AN, combining pharmaceutical and non-pharmaceutical strategies.

Given the complex nature of AN, a comprehensive assessment of existing treatments is crucial to inform clinical practice and enhance patient care. While numerous reviews have scrutinized specific therapeutic agents, there is a lack of thorough analyses comparing the effectiveness and safety of various treatment approaches across different patient groups. Such comparative research is crucial for determining the most efficacious interventions, particularly considering the condition’s strong link to metabolic syndromes and other systemic ailments. This systematic review aims to address the current knowledge deficit by assessing and contrasting the efficacy of diverse treatment modalities for acanthosis nigricans. The primary goal is to offer a detailed compilation of evidence from high-caliber clinical studies, concentrating on the effectiveness of these treatments in mitigating the dermatological manifestations of AN and tackling its root causes. Through an examination of the merits and drawbacks of various therapeutic strategies, this review endeavors to guide clinical decision-making and lay the groundwork for more effective and tailored management approaches for patients with AN.

## Methods

In line with a predetermined protocol registered in PROSPERO (CRD42023444441), this systematic review was undertaken. The review’s report adhered to the guidelines set forth in the Preferred Reporting Items for Systematic Reviews and Meta-Analysis checklist (21).

### Eligibility criteria

The study encompassed individuals of all ages diagnosed with mild to moderate Acanthosis Nigricans (AN) affecting the neck and axilla. The treatments under investigation included urea, glycolic acid (GA) peel, Trichloroacetic acid (TCA) peel, tretinoin, and salicylic acid. These interventions were also used as comparators. The research assessed outcomes using the Acanthosis Nigricans Area and Severity Index (ANASI) score, melanin (M) and erythema (E) indices measured by the narrowband reflectance spectrophotometer (Mexameter MX18), as well as investigator’s global evaluation (IGE) and participant’s global evaluation (PGE) scales. The study design employed was randomized controlled trials (RCTs) Table 1.

**Table 1:** Search strategy used to identify related studies using different databases.

Search query	Database
(“Acanthosis Nigricans” OR “AN”) AND (“topical treatment” OR “topical therapy” OR “cream” OR “ointment” OR “peeling” OR “chemical peel” OR “urea” OR “tretinoin” OR “glycolic acid” OR “trichloroacetic acid” OR “salicylic acid”) AND (“randomized controlled trial” OR “RCT”)	Pubmed (1,244)
	Scoups (101)
	Cochrane Library (233)
	WOS (4,826)
	ClinicalTrials.gov (3)
	Total (6,407)

### Exclusion criteria

The study excluded participants who met any of the following criteria: those with current or recent neck and axilla skin conditions or infectious diseases, individuals with severe medical issues (such as liver disease), patients who had received other topical treatments within 4 weeks of enrolment, those with photosensitivity, immunocompromised individuals, pregnant or breastfeeding women, people undergoing oral retinoid therapy, and those with neck or axilla tattoos. Additionally, non-randomized controlled trials and studies not published in English were omitted from consideration.

### Search strategy

A comprehensive literature review was conducted across multiple databases, including Medline, Embase, the Cochrane Central Register of Controlled Trials, Scopus, ClinicalTrials.gov, and Web of Science. The search encompassed all available records from the inception of each database until June 2024, with no date restrictions applied. The full details of the search methodology can be found in Supplementary material. To ensure thoroughness, the researchers also hand-searched the reference lists of included studies to identify any relevant RCTs that may have been overlooked during the initial systematic search.

### Data selection and extraction

The screening of titles and abstracts against eligibility criteria, assessment of full texts, and extraction of data from qualifying trials were conducted independently by two reviewers, working in parallel. Any disagreements were settled through consensus or by consulting a third reviewer prior to analysis. Data extraction was carried out using an Excel spreadsheet, with the following information collected from each eligible trial: the lead author’s name and publication year, study arms, participants’ gender and age, duration of follow-up, medication dosage, and reported outcomes of interest (ANASI and/or ANSC); melanin and erythema indices (M/E); IGE and PGE; and adverse events. In this review, IGE/PGE refer to global evaluation scales, while IGH/PGH were used specifically for hyperpigmentation outcomes.

### Risk-of-bias assessment

The risk-of-bias evaluation for the included RCTs was conducted independently by two assessors, utilizing the updated Cochrane risk-of-bias assessment tool. This tool examines six primary potential sources of bias: the randomization procedure, departures from the intended intervention, absent outcomes, outcome measurement, and selection of reported results. The assessors categorized studies as having a high risk of bias, some concerns, or a low risk of bias (22).

## Results

### Study selection

**Figure 1:**
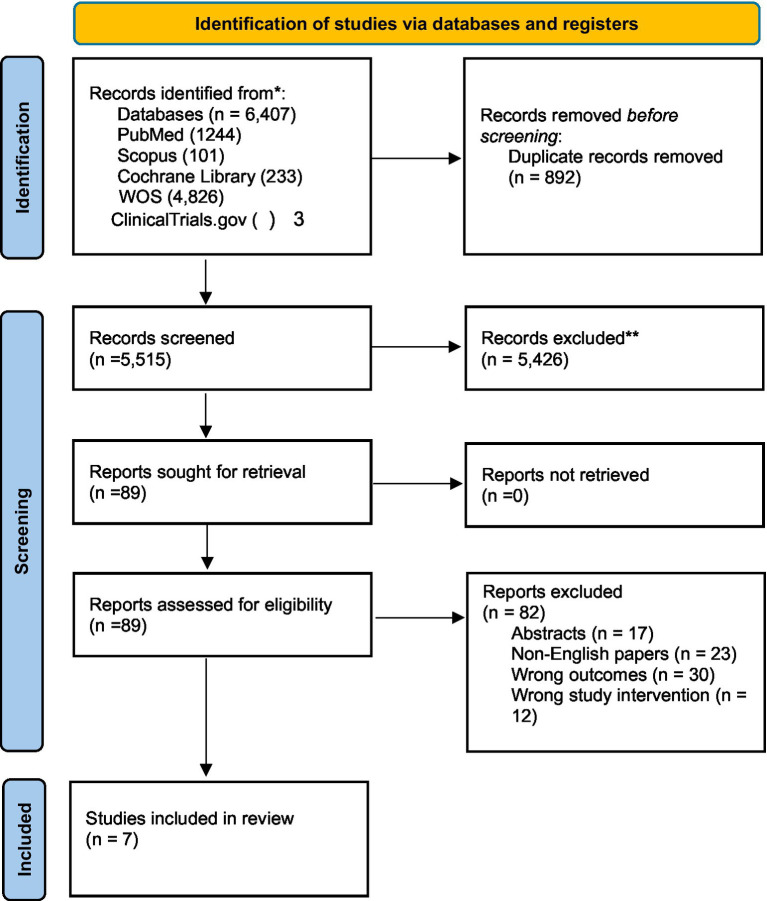
PRISMA flow diagram of the selection process.

A comprehensive search of multiple databases yielded 6,407 results: 1,244 from PubMed, 101 from Scopus, 3 from ClinicalTrials.gov, 4,826 from Web of Science, and 233 from the Cochrane Library. Following the removal of 892 duplicates, 5,515 articles underwent screening based on their titles and abstracts. This process identified 89 potentially relevant articles for full-text evaluation. After thorough assessment, seven articles met the inclusion criteria and were subsequently included in the systematic review (23–29) (seven studies; see Figure 1).

### Baseline characteristics

This review included seven randomized controlled trials with 268 patients in total. Each study had between 30 and 41 participants and evaluated different topical treatments for AN. A summary of their fundamental characteristics is presented in Table 2. The neck and axilla were the primary treatment areas, with some investigations also addressing lesions on the back, face, and elbows. Therapeutic interventions included topical applications such as urea (10–20%), tretinoin (0.025–0.05%), salicylic acid (10%), glycolic acid (35–70%), and trichloroacetic acid (15%), administered twice per day or at defined intervals over a period of 8 weeks to 2 months. Women constituted the majority of participants across all studies. The average age spanned from 12.8 to 36.4 years, with body weights ranging from 66.7 to 95.5 kg, and BMI values between 28.9 and 36.1 kg/m^2^. The predominant Fitzpatrick skin phototypes were Type IV and V, although some studies also included Type III.

**Table 2:** Baseline characteristics of included studies.

Author, year	Study design	Type of treatment	Region of the lesion (Neck, Axilla)	Unilateral/bilateral	Sample size	Treatment regimen			Gender		Age (years) (mean, SD)	Weight (kg) (mean, SD)	Body mass index (kg/m^2^) (mean, SD)	Fitzpatrick skin phototype: *n* (%)
						Dosage	Frequency	Duration	Male	Female				
Treesirichod A, 2021 (23)	RCT	1. 10% Urea 2. 0.025% tretinoin	Neck, and back	Bilateral	38	1. 10% Urea 2. 0.025% tretinoin	Twice daily	8 weeks	30%	70%	31.2 ± 14.4 years	86.3, 16.7	NR	1. Urea: Type III 0 (0%) Type IV 15 (78.9%) Type V 4 (21.1%) 2. Tretinoin: Type III 2 (10.5%) Type IV 6 (31.6%) Type V 11 (57.9%)
Treesirichod A, 2023 (24)	RCT	1. Salicylic acid 2. Urea	Posterior neck	Each modality on a side	39	1. 10% Salicylic acid 2. 10% Urea	Twice daily	8 weeks	Salicylic acid 73.6% Urea 50%	Salicylic acid 26.3% Urea 50%	Salicylic acid 13.74 (1.79) Urea 14.95 (1.67)	Salicylic acid 88.86 (26.12) Urea 86.91 (20.99)	Salicylic acid Overweight 2 (10.5%) Obese 17 (89.5%) Urea Overweight 5 (25.0%) Obese 15 (75.0%)	Salicylic acid Type IV 7 (36.8%) Type V 12 (63.2%) Urea Type IV 10 (50.0%) Type V (50.0%)
Rajegowda H, (29)	RCT	1. Tretinoin 0.025% 2. 15% Trichloroacetic acid	NR	NR	41	1. Tretinoin 0.025% 2. 15% trichloroacetic acid	1. Tretinoin daily 2. Trichloroacetic acid once in 15 days	2 months	1. Tretinoin >2 2. Trichloroacetic acid >4	1. Tretinoin >23 2. Trichloroacetic acid >21	NR	NR	NR	NR
Ghiasi M, 2024 (25)	RCT	1. Tretinoin 0.05% 2. Glycolic acid 70%	Neck, axilla	Each modality on a side	30	1. Tretinoin 0.05 2. Glycolic acid 70%	1. Tretinoin 0.05 every other night 2. Glycolic acid 70% every 2 weeks	2 months	70%	30%	25.27 ± 10.74	NR	29.67 ± 4.92	1–2: 3 (10%) 2–3: 25 (83.3%) 3–4: 2 (6.7%)
Treesirichod A, 2021 (23)	RCT	1. Urea 10% 2. Urea 20%	Neck, back	Each modality on a side	40	1. Urea 10% 2. Urea 20%	Twice a day	2 months	1. Urea 10%: 11 (55%) 2. Urea 20%: 13 (65%)	1. Urea 10%: 9 (45%) 2. Urea 20%: 7 (35%)	1. Urea 10%: 12.8 ± 0.4 2. Urea 20%: 12.6 ± 0.5	1. Urea 10%: 70.3 ± 24.5 2. Urea 20%: 66.7 ± 16.3	1. Urea 10%: 31.7 ± 12.7 2. Urea 20%: 28.9 ± 4.1	1. Urea 10% Type III 1 (5.0%) Type IV 11 (55.0%) Type V 8 (40.0%) 2. Urea 20% Type III 2 (10.0%) Type IV 9 (45.0%) Type V 9 (45.0%)
Kritsanaviparkporn C, 2022 (28)	RCT	1. Tretinoin 0.025% 2. Tretinoin 0.05%	Posterior neck	Each modality on a side	40	1. Tretinoin 0.025% 2. Tretinoin 0.05%	Once every other day during the first 2 weeks, and then once daily after that	2 months	1. Tretinoin 0.025%: 3(15%) 2. Tretinoin 0.05%: 3 (15%)	1. Tretinoin 0.025%: 17 (85%) 2. Tretinoin 0.05%: 17 (85%)	1. Tretinoin 0.025%: 36.05 (12.20) 2. Tretinoin 0.05%: 36.40 (12.06)	1. Tretinoin 0.025%: 93.26 (12.90) 2. Tretinoin 0.05%: 95.47 (16.43)	1. Tretinoin 0.025%: 35.59 (3.62) 2. Tretinoin 0.05%: 36.07 (6.34)	1. Tretinoin 0.025%: Type III 1 (5%) Type IV 10 (50%) Type V 9 (45%) 2. Tretinoin 0.05%: Type III 1 (5%) Type IV 10 (50%) Type V 9 (45%)
Bharati B, 2024 (27)	RCT	1. 15% trichloroacetic acid (TCA) 2. 35% glycolic acid	Neck, face, elbow	Each modality on a side	40	1. 15% trichloroacetic acid (TCA) 2. 35% glycolic acid	3 peeling sessions at 2 weeks interval	2 months	1. 15% trichloroacetic acid: 10 (50.0%) 2. 35% glycolic acid: 9 (45.0%)	1. 15% trichloroacetic acid: 10 (50.0%) 2. 35% glycolic acid: 11 (55.0%)	1. 15% trichloroacetic acid: 32.40 ± 11.52 2. 35% glycolic acid: 29.05 ± 9.12	NR	NR	NR

### M index (neck and axilla)

The M index, a measure of hyperpigmentation severity in AN, exhibited diverse responses to various treatments. Research by Treesirichod et al. (23) indicated that urea 10% decreased the neck M index from 572.7 to 539.6, while tretinoin 0.025% achieved a more significant reduction from 664.5 to 529.7, suggesting tretinoin’s superior efficacy. These findings were supported by Rajegowda et al. (29), who demonstrated tretinoin’s greater effectiveness compared to urea in diminishing neck hyperpigmentation. Likewise, Kritsanaviparkporn et al. (28) observed that tretinoin 0.05% markedly lowered the axilla M index, with urea showing less pronounced improvements. These outcomes underscore tretinoin’s enhanced capacity to alleviate hyperpigmentation severity in AN relative to urea. The more substantial improvements noted with tretinoin, particularly in the neck area, indicate its potential as a more potent treatment option for these regions. Nevertheless, urea still offers therapeutic advantages, especially in less severe cases. A separate investigation by Treesirichod et al. (23) revealed that urea at 20% concentration led to a more substantial reduction in the neck M index compared to 10% urea (e.g., 176.7 vs. 85.9 at 8 weeks), highlighting urea’s dose-dependent effect on skin thickening. Furthermore, salicylic acid, examined by Treesirichod et al. (24), also reduced the M index, albeit at a slower rate than urea-based treatments.

### E index (neck and axilla)

The assessment of skin texture enhancement, as measured by the E index, showed significant variations between urea and tretinoin treatments. Research by Treesirichod et al. (23) indicated that 20% urea markedly improved the E index for both neck and axilla areas, while tretinoin exhibited more substantial improvements in the neck region but less pronounced effects on the axilla. Similarly, Rajegowda et al. (29) observed that urea was more effective than tretinoin in enhancing skin texture on the axilla. These studies collectively suggest that although tretinoin is efficacious in addressing hyperpigmentation, urea, especially at higher concentrations, may be the preferred option for improving skin texture in AN. Tretinoin remains effective but demonstrates a slightly slower rate of progress compared to urea.

### Improvement grade of hyperpigmentation (IGH) and percentage of generalized hyperpigmentation (PGH)

Some included trials additionally employed IGH and PGH as specific measures of hyperpigmentation improvement. The effectiveness of tretinoin in improving hyperpigmentation consistently surpassed that of urea, as evidenced by IGH and PGH scores, which capture the extent and degree of pigment reduction. A study by Treesirichod et al. (23) revealed that tretinoin 0.05% yielded a 90% enhancement in IGH and a 75% enhancement in PGH, while urea achieved 75 and 50% improvements, respectively. Corroborating these findings, Rajegowda et al. (29) noted that tretinoin 0.05% outperformed urea 10% in both IGH and PGH improvements. Further substantiation came from Kritsanaviparkporn et al. (28), who demonstrated tretinoin’s superior efficacy in diminishing generalized hyperpigmentation. These outcomes support tretinoin’s potential as the preferred treatment for reducing the severity and extent of hyperpigmentation in AN.

### ANASI and ANSC scores

The ANASI score, which evaluates severity, and ANSC, which assesses compliance, were primarily reported in studies evaluating salicylic acid and urea. Treesirichod et al. (24) found that both treatments reduced ANASI scores significantly at week 8. However, urea demonstrated a slightly better compliance rate compared to salicylic acid, as evidenced by fewer reports of adverse effects such as dryness and peeling. Both salicylic acid and urea effectively reduce ANASI scores, but urea appears to be better tolerated.

### Patient-reported satisfaction

Treatment efficacy varied in terms of patient satisfaction. According to Treesirichod et al. (23), tretinoin yielded high levels of contentment, with 47.4% of subjects experiencing 75–90% improvement after 8 weeks. Urea also demonstrated positive outcomes, albeit with a lower percentage of participants reporting satisfaction above 75% improvement. Ghiasi et al. (25) noted that tretinoin resulted in greater satisfaction for axillary AN cases, while glycolic acid showed limited efficacy, with fewer subjects indicating excellent improvement scores. Across studies, tretinoin consistently garnered higher patient satisfaction, particularly in more complex presentations such as axillary AN.

### Grade of improvement

Across studies, tretinoin consistently outperformed other treatments, with a higher percentage of patients achieving >75% improvement. For example, in the study by Rajegowda et al. (29), tretinoin led to >50% improvement in 71.43% of participants compared to 26.3% with salicylic acid (24). Glycolic acid showed the least improvement, with most participants only achieving <50% improvement (25). Among all treatments, tretinoin showed the greatest improvement rates, though urea and salicylic acid remained effective alternatives with fewer side effects.

### Adverse events

Research has documented a range of adverse effects, with varying frequencies and severities. Treesirichod et al. (23) reported that tretinoin, especially at higher concentrations (0.05%), frequently caused skin dryness, irritation, and peeling, particularly in the neck region. While generally mild, these side effects occasionally led to treatment discontinuation. In comparison, urea exhibited fewer adverse reactions, with rare instances of mild stinging or irritation. This observation was supported by Rajegowda et al. (29), who noted that urea was better tolerated than tretinoin. Additional evidence came from Kritsanaviparkporn et al. (28), who observed more frequent and severe peeling with tretinoin usage, whereas urea only induced minor skin dryness. Although tretinoin demonstrated greater effectiveness, its higher likelihood of adverse events necessitates vigilant monitoring, especially in individuals with sensitive skin.

Among the other evaluated topical treatments, Treesirichod et al. (24) reported that 10% salicylic acid caused only mild and short-lived adverse events, mainly dryness (10%), peeling (26%), and burning (5%), which resolved without complications. Kritsanaviparkporn et al. (28) found that both concentrations of tretinoin (0.025% vs. 0.05%) showed similar safety profiles, with mild to moderate erythema, dryness, peeling, itching, and finally burning or stinging which was sometimes bothersome enough to pause treatment briefly, though the overall safety was similar between groups. Ghiasi et al. (25) observed no adverse events with 70% glycolic acid peeling, which was well tolerated, whereas tretinoin caused mild erythema and scaling. For trichloroacetic acid (TCA) peels, Rajegowda et al. (29) reported fewer side effects than with tretinoin, including erythema, burning, desquamation, and occasional post-inflammatory hyperpigmentation. In contrast, Bharati et al. (27) noted higher rates of peeling (up to 70%) and burning (up to 45%), along with erythema and PIH, although these were mild, transient, and did not lead to dropouts. Overall, adverse events across these topical therapies were mild, short-lived, and manageable.

In summary, both urea and tretinoin demonstrated efficacy in treating Acanthosis Nigricans, but their outcomes differed based on the specific treatment goals. Tretinoin consistently outperformed urea in reducing hyperpigmentation (M index, IGH, and PGH), making it the preferred option for addressing pigmentation severity and extent. However, urea was more effective in improving skin texture (E index) and was associated with fewer adverse events, suggesting its suitability for patients prioritizing tolerability or seeking improvements in skin texture. Other topical options, including glycolic acid, salicylic acid and trichloroacetic acid, showed generally mild and self-limited adverse events, supporting their role as alternative or adjunctive therapies in selected cases (Table 3).

**Table 3:** Summary of outcomes.

Author, year	Measured outcomes (Mean, 95% CI)	Measured *p* values	Adverse events	Previous treatment: *n* (%)	Compliance: mean (SD)	Result
Treesirichod A, 2019	Urea: M index (Neck: Week 0–8), E index (Neck: Week 0–8), M index (Back: Week 0–8), E index (Back: Week 0–8); Tretinoin: M & E index for Neck and Back at Weeks 0–8.	M index (neck): *p* < 0.001 (favoring tretinoin) E index (neck): *p* = 0.984 M index (back): *p* = 0.116 E index (back): *p* = 0.687 IGE (wk8): *p* = 0.002 (favoring tretinoin) PGE (wk8): *p* = 0.001 (favoring tretinoin) Peeling (wk2): *p* = 0.041 (more with tretinoin)	Erythema, Burning, Hypopigmentation, P. I. H., Skin Peeling	Urea: 36.8 > 75% improvement; Tretinoin: 10.5 > 90% improvement	N/A	Significant improvement in IGE and PGE scores at 8 weeks, Tretinoin showed higher improvement percentages.
Treesirichod A, 2023 (24)	Salicylic acid: M index at Week 0–8, Urea: M index at Week 0–8. IGE and PGE scores of treatment at Week 0–8.	M index (neck): *p* = 0.114 E index (neck): *p* = 0.721 M index (back): *p* = 0.286 E index (back): *p* = 0.580 ANSC (neck): *p* = 0.105 IGE / PGE: NS (no significant difference)	Dryness (10.5%), slight irritation	N/A	N/A	Urea showed consistent improvement in M index, with slight irritation reported for Salicylic acid at Week 2.
Rajegowda H, (29)	Tretinoin & Trichloroacetic acid: Improvement rates for 25%, 26–50%, and 51–75%.	Pigmentation improvement: *p* = 0.0395 (favoring tretinoin) Adverse events: more with tretinoin (no *p*-value reported)	N/A	Tretinoin: 28.57 > 25% improvement; Trichloroacetic acid: 10 > 25% improvement	N/A	Trichloroacetic acid showed higher improvement in the 26–50% range, with a few adverse events.
Ghiasi M, 2024 (25)	Tretinoin (Neck and Axillary), Glycolic acid (Neck and Axillary): Poor to Excellent improvement rates.	Neck – response: *p* = 0.058 Neck – satisfaction: *p* = 0.200 Axilla – response: *p* = 0.021 Axilla – satisfaction: *p* = 0.008	Visible irritation, slight skin issues	N/A	60% compliance reported	Tretinoin showed more significant improvement in both Neck and Axillary regions compared to Glycolic acid.
Treesirichod A, 2021 (23)	Urea 10% and Urea 20%: M & E index differences at Week 0–8. IGE and PGE improvement rates for both Urea treatments at 8 weeks.	M index (neck): *p* = 0.001 E index (neck): *p* = 0.530 M index (back): *p* = 0.190 E index (back): *p* = 0.818 IGE (wk2–8): *p* = 0.005 → 0.000 PGE (wk2–8): *p* = 0.000 → 0.001	N/A	Urea 10%: 75 > 50% improvement; Urea 20%: 45 > 75% improvement	N/A	Significant improvement in both IGE and PGE scores for Urea treatments, with Urea 20% showing higher results.
Kritsanaviparkporn C, 2022 (28)	Tretinoin 0.025 and 0.05% M & E index differences at Week 0–8. IGE and PGE improvement rates for both concentrations at 8 weeks.	M index (neck): *p* = 0.243 E index (neck): *p* = 0.943 ANSC (neck): *p* = 0.070 IGE (wk2): *p* = 0.045* IGE (wk4–8): >0.05 PGE (wk2–8): >0.05 Irritation scores: >0.15 (*only significant difference at week 2)	Mild dryness and irritation (10.5%)	Tretinoin 0.025%: 100% improvement for 5 participants	N/A	Significant improvement in both 0.025 and 0.05% Tretinoin, with the 0.05% concentration showing higher IGE improvements.
Bharati B, 2023 (27)	Tretinoin 0.025%: M index for Improvement Rate of 55% (Week 0–8).	ANASI (within groups): *p* < 0.001 (both) ANASI (between groups): *p* < 0.001 (favoring TCA) PAS (wk8): *p* < 0.001 (favoring TCA) PSS (wk8): *p* = 0.039 (favoring TCA)	N/A	N/A	N/A	Significant improvement in M index with 55% improvement rate at Week 8 for Tretinoin 0.025%.

## Discussion

AN is characterized by velvety, darkened, thickened skin, primarily occurring in the neck, armpit, and groin folds. AN can present as a clinical condition linked to various genetic disorders, many stemming from insulin resistance syndromes or FGFR functional abnormalities (30–32). This comprehensive review aimed to thoroughly evaluate and contrast the effectiveness of diverse topical treatments for AN, with a specific focus on urea and tretinoin. Despite the availability of numerous therapies, understanding their comparative efficacy and safety profiles is essential for helping physicians tailor interventions to diverse patient needs. This investigation sought to address this gap by synthesizing data from high-quality RCTs to assess these medications’ performance across multiple parameters, including lesion severity, redness reduction, and scar improvement.

The systematic review examined seven RCTs that satisfied the inclusion criteria. These investigations evaluated the therapeutic effectiveness of urea and tretinoin in addressing AN-related outcomes. The results consistently showed that tretinoin outperformed urea in diminishing AN severity indicator, as gaged by metrics such as M index, Investigator’s Global Evaluation (IGE), and Patient’s Global Evaluation (PGE). Additionally, tretinoin exhibited marked enhancements in nodular severity and scarring, as evidenced by improved ANASI and ANSC scores. Nonetheless, urea proved more efficacious in alleviating erythema (E index), particularly when administered at higher concentrations. The adverse event profiles varied considerably between the two agents: tretinoin was linked to a higher frequency of irritation, peeling, and dryness, while urea displayed better tolerability with fewer and less severe side effects. These observations highlight the importance of customizing treatment plans to suit individual patient characteristics and therapeutic objectives, as the selection of agent involves balancing efficacy against tolerability.

Tretinoin, a vitamin A derivative, operates through various mechanisms to achieve its therapeutic effects (33–36). By accelerating epidermal renewal, tretinoin minimizes the build-up of dead skin cells, thus inhibiting comedone formation. Moreover, its anti-inflammatory characteristics aid in alleviating redness and post-inflammatory hyperpigmentation (37). Nevertheless, the potency of tretinoin also explains its higher frequency of side effects, including skin flaking and irritation, particularly when used at 0.05% concentration (28, 38).

Urea serves as both a keratolytic and humectant, improving skin moisture and aiding in keratin breakdown (39, 40). These characteristics make it particularly useful in alleviating redness and enhancing skin quality. Multiple studies have demonstrated urea’s superior effectiveness in reducing the E index, underscoring its role as an anti-erythematous compound. Its mode of action is especially advantageous for individuals with sensitive skin or those primarily seeking to diminish redness associated with AN (23, 24, 26). The varied outcomes observed in this analysis can be attributed to these specific mechanisms, emphasizing the importance of tailoring treatment approaches to individual clinical presentations and patient preferences.

Trichloroacetic acid (TCA) is a superficial chemical exfoliant that induces epidermal damage, followed by healing and regeneration. TCA is corrosive and induces coagulation of skin proteins, resulting in icing. Protein precipitation results in necrosis and epidermal degradation, thereby inciting inflammation and the activation of wound healing systems. This results in re-epithelialization and the restoration of smoother skin (41–43). The benefits of TCA include its stability, which allows for a correlation between systemic absorption and peel depth with the degree of frost, making the endpoint easier to assess (41).

Beyond urea and tretinoin, other topical therapies also showed useful effects in randomized trials. Salicylic acid 10% produced improvements comparable to urea, with tolerability advantages such as only mild dryness or peeling (24). Glycolic acid (35–70%) peel was less effective overall, especially compared to tretinoin, and patient satisfaction was lower (25, 27). In contrast, trichloroacetic acid (15%) peel outperformed glycolic acid on both ANASI scores and patient satisfaction, although higher concentrations were associated with more peeling and burning (27). Taken together, these findings suggest that tretinoin remains the most effective for reducing hyperpigmentation, urea provides a dose-responsive benefit for texture and erythema, salicylic acid can serve as a mild and tolerable alternative, and TCA peel is preferable over glycolic acid when a procedural option is chosen.

Several limitations of this systematic review warrant consideration. The studies included had relatively modest sample sizes, which diminished the statistical robustness of the results and restricted their broader applicability. The diversity in treatment protocols, encompassing variations in concentrations, application frequencies, and durations, hindered direct comparisons of effectiveness and safety outcomes. Moreover, the predominance of participants with Fitzpatrick skin types IV and V constrains the relevance of the findings to individuals with lighter or darker complexions. The studies primarily assessed short-term outcomes, spanning 8 weeks to 2 months, leaving uncertainties regarding the long-term efficacy and safety profiles of these treatments. Lastly, the emphasis on single-agent therapies may have overlooked potential synergistic benefits of combining urea or tretinoin with other substances, such as benzoyl peroxide or antibiotics.

Notwithstanding its constraints, this review exhibits numerous strengths that bolster its value to the current scientific literature. A comprehensive and meticulous search methodology was implemented, spanning multiple databases and resulting in a substantial dataset for examination. The review’s focus on RCTs ensures a high standard of evidence, reducing bias and improving the credibility of the results. The evaluation of various outcome measures, including M index, E index, IGE, PGE, ANASI, and ANSC, offers a comprehensive assessment of urea and tretinoin’s effectiveness across different clinical metrics. Across the included trials, adverse events were generally mild, transient, and manageable, further supporting the safety profile of the evaluated topical treatments for AN.

Looking ahead, several areas warrant further investigation. Future research should focus on evaluating the sustained efficacy and safety of urea and tretinoin over extended periods, providing insights into their long-term benefits and risks. Exploring the synergistic effects of combining urea or tretinoin with other therapeutic agents could yield optimized treatment regimens for complex cases. Studies involving participants with diverse skin types, age groups, and severities would enhance the generalizability of the findings. Advances in drug delivery systems, such as nanotechnology, offer the potential to improve the efficacy and tolerability of these treatments. Additionally, evaluating the economic implications of urea and tretinoin treatments could inform healthcare policies and improve access to effective AN therapy.

## Conclusion

This comprehensive review highlights the effectiveness of urea and tretinoin in treating AN, with each agent offering unique benefits. Urea, known for its moisturizing and keratolytic qualities, presents a well-tolerated and efficient solution for patients experiencing erythema or mild hyperkeratosis. Conversely, tretinoin, with its robust keratolytic and depigmenting actions, is more appropriate for moderate to severe cases where skin discolouration and hyperkeratosis are the primary issues. However, tretinoin’s increased likelihood of causing irritation and skin peeling requires careful patient supervision and customized application protocols. In addition, other topical agents such as glycolic acid, salicylic acid, and trichloroacetic acid have demonstrated acceptable safety profiles with only mild, transient adverse events. Subsequent research should aim to refine concentration levels and explore combination therapies to boost efficacy while reducing adverse reactions. This analysis emphasizes the significance of tailored treatment strategies in achieving the best possible results for individuals with AN.

## Data Availability

The original contributions presented in the study are included in the article/Supplementary material, further inquiries can be directed to the corresponding author.
