# Supplementary material for: The efficacy of topical treatments for acanthosis nigricans: a systematic review of randomized controlled trials
Source: Front Med (Lausanne). 2025 Oct 10;12:1641322. doi: 10.3389/fmed.2025.1641322 (PMC12549304; doi:10.3389/fmed.2025.1641322)
Supplement: Supplementary file 1 [file Supplementary_file_1.pdf]

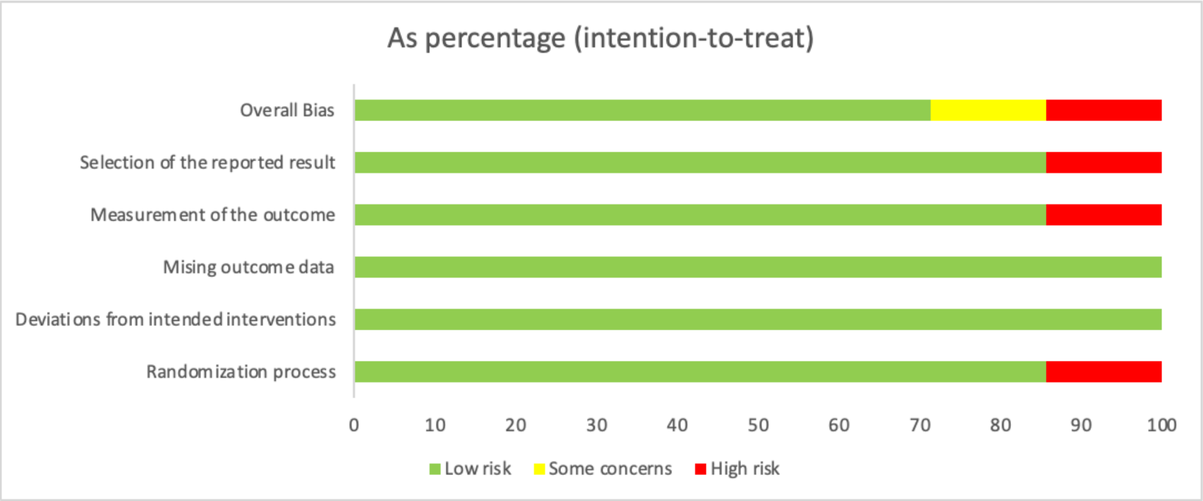

figure S1: bar chart for Risk of bias assessment

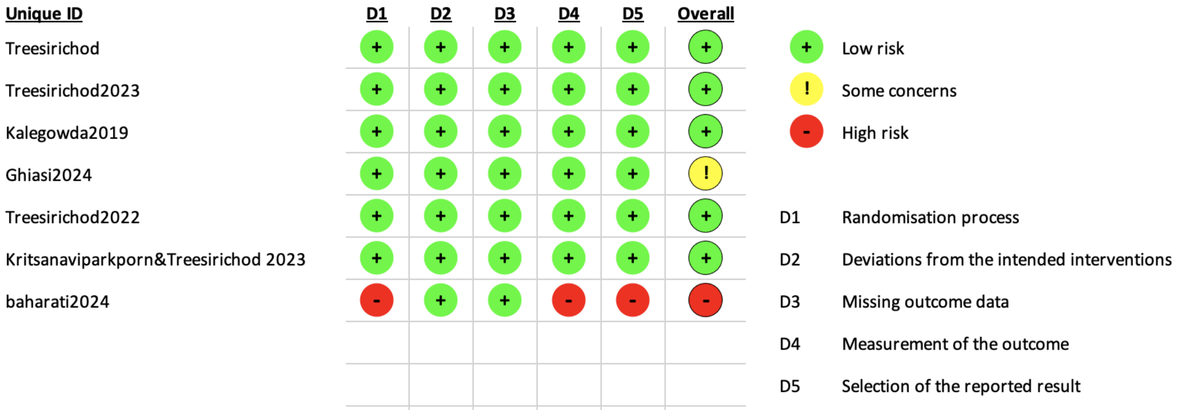

figure S2: traffic light chart for Risk of bias assessment
